# Electric Field Controlled Indirect-Direct-Indirect Band Gap Transition in Monolayer InSe

**DOI:** 10.1186/s11671-019-3162-0

**Published:** 2019-10-15

**Authors:** Xian-Bo Xiao, Qian Ye, Zheng-Fang Liu, Qing-Ping Wu, Yuan Li, Guo-Ping Ai

**Affiliations:** 1 School of Computer Science, Jiangxi University of Traditional Chinese Medicine, Nanchang, 330004 China; 2 School of Science, East China Jiaotong University, Nanchang, 330013 China; 3 Department of Physics, Hangzhou Dianzi University, Hangzhou, 310018 China

**Keywords:** Monolayer InSe, Electric field, Indirect-direct-indirect band gap transition

## Abstract

Electronic structures of monolayer InSe with a perpendicular electric field are investigated. Indirect-direct-indirect band gap transition is found in monolayer InSe as the electric field strength is increased continuously. Meanwhile, the global band gap is suppressed gradually to zero, indicating that semiconductor-metal transformation happens. The underlying mechanisms are revealed by analyzing both the orbital contributions to energy band and evolution of band edges. These findings may not only facilitate our further understanding of electronic characteristics of layered group III-VI semiconductors, but also provide useful guidance for designing optoelectronic devices.

## Introduction

Since the pioneering work on the experimental realization of a single-layer graphite, namely graphene [1, 2], atomically thin two-dimensional (2D) materials have been paid lots of attentions [3, 4]. Various monolayer 2D materials have been theoretically predicted or experimentally discovered including silicene [5–7], germanane [8], black phosphorus [9, 10], transition metal dichalcogenides (TMDs) [11–13], and hexagonal boron nitride [14–16]. Although these atomically thin 2D materials have the similar honeycomb lattice structures, their electronic structures and conductivity properties are quite different including metal [1, 2, 5–8], semiconductor [9–13], and insulator [14–16]. Therefore, according to their electronic characters, these single layer 2D materials may find applications in the design of multifunctional electronic and optical devices [3, 4]. For example, tunable optical devices with high-quality factor based on Si-graphene metamaterials [17], Cu-graphene metamaterials [18], and MoS_2_-SiO_2_-Si waveguide structures [19] are proposed. Perfect valley or/and spin polarization devices based on the ferromagnetic graphene [20], strained graphene with Rashba spin-orbit coupling and magnetic barrier [21], and strained silicene with an electric field are suggested [22, 23]. Moreover, the interaction effects between the decomposition components of SF_6_ and different materials including N-doped single-wall carbon nanotubes [24], Pt_3_-TiO_2_(1 0 1) surface [25], Ni-doped MoS_2_ monolayer [26], and Pd (1 1 1) surface [27] are investigated by using the density functional theory (DFT).

Group III–VI compounds MXs (M = Ga, In and X = S, Se, Te) are another family of layered 2D materials. Due to their unique electrical characters, these materials have drawn many researchers’ attentions [28]. DFT [29–33] and tight-binding model [34] calculations show that energy band gap of layered MXs is thickness dependent, increasing from 1.3 to 3.0 eV as the number of layers is decreased. At the same time, direct-indirect band gap transition is observed, which is opposite to the behaviors of layered black phosphorus [9, 10] and TMDs [11–13]. This sizable energy band gap modulation of layered MXs may be used to design optoelectronic devices [35, 36]. In addition, the stability of InSe doped with oxygen defects is investigated and found that it is more stable than black phosphorus in the air [37]. The magnetism of InSe monolayer can be tuned by adsorbing As [38], C, and F [39]. Huge spin-charge conversion effect is found in bilayer InSe due to the broken mirror symmetry [40]. Moreover, the electronic structure and the current-voltage characteristics of monolayer InSe nanoribbons strongly depend on the edge states [41]. On the other hand, experimental researches verify the layer-dependent electronic structures of MXs and they can responds to the light spanning the visible and near-infrared regions [42–45]. Also, the carrier mobilities of MXs are found to be high, enabling that they may be used to design field effect transistors. For bulk GaS and GaSe, the carrier mobilities are about 80 and 215 cm ^2^ V ^−1^ S ^−1^ [46], respectively. For the monolayer InSe, the carrier mobility is even up to almost 10 ^3^ cm ^2^ V ^−1^ S ^−1^ [47]. Moreover, band gap of layered InSe can be manipulated by uniaxial tensile strain, which is identified by the photoluminescence spectra [48].

From the viewpoint of the optoelectronic device designment, the efficiency of the devices based on direct band gap semiconductors are better than those based on indirect band gap ones. Therefore, transforming indirect band gap few-layer MXs to direct band gap type is a challenge for scientific community. Very recently, band gap manipulation and indirect-direct band gap transition are found in monolayer InSe by uniaxial strain [49]. Also, direct band gap semiconductors have been obtained by stacking 2D n-InSe and p-GeSe(SnS). And the band gap values and band offset of these van der Waals heterojunctions can be tuned by the interlayer coupling and external electric field [50]. In addition, the possible stacking configurations of bilayer InSe and the influence of the perpendicular electric field on their electronic structures are studied. Indirect band gap bilayer InSe can be transformed to the metallic type by varying the electric field strength [51]. Similarly, in other buckled 2D materials like silicene [52], germanene [53], transition metal dichalcogenides [54, 55], and black phosphorus [56], a perpendicular electric field is also proposed to tune their band gap and electronic characteristics. In light of these previous studies, a natural question may be inquired what are the electric field effects on the electronic structures of the monolayer InSe.

In this letter, the effects of a perpendicular electric field on the electronic structures of the monolayer InSe are investigated by using the tight-binding model Hamiltonian. Indirect-direct-indirect band gap transition can be achieved in the considered system with increasing electric field strength. At the same time, band gap of the monolayer InSe is decreased gradually, eventually rendering it metallic. The underlying physics mechanisms of these effects are unraveled by analyzing the orbital decomposition for the energy band and the electric field-modulated energy position shift of the band edges. Our studies may benefit to fundamentally understand the electronic properties of few-layer InSe as well as provide theoretical bases for 2D optoelectronic devices.

## Methods

**Fig. 1:**
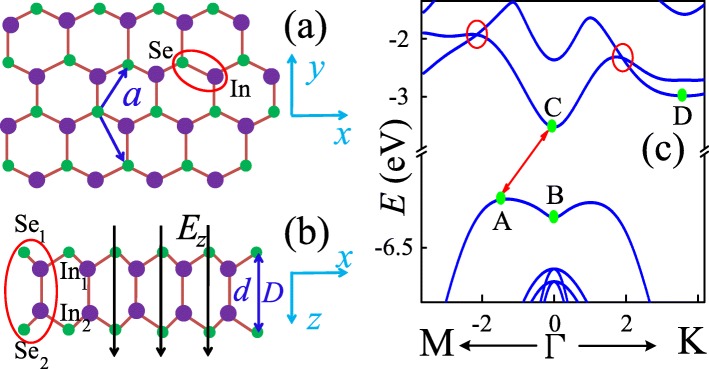
(Color online) Top (**a**) and side (**b**) view of the monolayer InSe in the *xy* and *xz* planes, respectively. The lattice constant between the nearest In or Se ions in the *xy* plane is *a*, and the distance between the nearest In (Se) ions in different sublayers is *d* (*D*). A perpendicular electric field along *z*-axis *E*_*z*_ is applied to the monolayer InSe. **c** Energy band of monolayer InSe

The top view of InSe monolayer is sketched in Fig. 1a, where the big purple spheres represent indium ions while the small green ones depict selenium ions. This two types of ions form graphene-like hexagonal structure in the *xy* plane with lattice constant *a*, the distance between the nearest In or Se ions. Figure 1b shows the schematic of side view of InSe monolayer. Differing from graphene, two sublayers with mirror symmetry in the *xz* plane are observed. The vertical distance between In (Se) ions of different sublayers is set at *d* (*D*). Therefore, a unit cell of monolayer InSe consists of four ions *Se*_1_, *In*_1_, *Se*_2_, and *In*_2_, as shown by the red ellipse in Fig. 1b, in which number 1 (2) indicates the sublayer index.

The tight-binding Hamiltonian up to second-nearest neighbor interactions including all possible hoppings between the *s* and *p* orbitals of In and Se ions reads [34]

1 $$ H=\sum\limits_{l} H_{0l}+H_{ll}+H_{ll'},  $$

in which the sum runs over the sublayers *l*=1 and 2, and *l*^′^=2(1) as *l*=1(2). *H*_0*l*_, *H*_*ll*_, and $\phantom {\dot {i}\!}H_{ll^{\prime }}$ consist of terms coming from the on-site energies, hopping energies within and between the two sublayers, respectively. And the explicit expressions of them are given as [34]

2 $$\begin{array}{@{}rcl@{}} H_{0l}=\sum\limits_{i}[\varepsilon_{\text{In}_{s}}a_{lis}^{\dag}a_{lis}+ \sum\limits_{\alpha}\varepsilon_{\text{In}_{p_{\alpha}}}a_{{lip}_{\alpha}}^{\dag}a_{{lip}_{\alpha}}+ \\ \varepsilon_{\text{Se}_{s}}b_{lis}^{\dag}b_{lis}+ \sum\limits_{\alpha}\varepsilon_{\text{Se}_{p_{\alpha}}}b_{{lip}_{\alpha}}^{\dag}b_{{lip}_{\alpha}}], \end{array} $$

where the sum runs over all unit cells in sublayer *l*. $\phantom {\dot {i}\!}\varepsilon _{\mathrm {In(Se)}_{s}}$ is the on-site energy for the *s* orbital of In (Se) ions, while $\phantom {\dot {i}\!}\varepsilon _{\mathrm {In(Se)}_{p_{\alpha }}}$ is that for orbital *p*_*α*_ (*α*=*x*,*y*,*z*). $a_{lis}^{\dag }$ (*a*_*lis*_) is the creation (annihilation) operator for an electron in *s* orbital on In ions in unit cell *i* and sublayer *l*, but $\phantom {\dot {i}\!}a_{{lip}_{\alpha }}^{\dag }$ ($\phantom {\dot {i}\!}a_{{lip}_{\alpha }}$) for an electron in *p*_*α*_ orbital. Similarly, *b*^*†*^ (*b*) is the creation (annihilation) operator for an electron in the relevant orbital on Se ions.

3 $$\begin{array}{@{}rcl@{}} H_{ll}=H_{ll}^{(\text{In}-\text{Se})_{1}}+H_{ll}^{\text{In}-\text{In}}+H_{ll}^{\text{Se}-\text{Se}}+H_{ll}^{(\text{In}-\text{Se})_{2}}, \end{array} $$

in which [34]

4 $$ {{}{\begin{aligned} H_{ll}^{(\text{In}-\text{Se})_{1}}=\sum\limits_{<\text{In}_{li},\text{Se}_{lj}>}\{T_{ss}^{(\text{In}-\text{Se})_{1}}b_{ljs}^{\dag} a_{lis}+T_{sp}^{(\text{In}-\text{Se})_{1}}\sum\limits_{\alpha}R_{\alpha}^{\text{In}_{li}\text{Se}_{lj}} \\ b_{ljp_{\alpha}}^{\dag} a_{lis}+T_{ps}^{(\text{In}-\text{Se})_{1}}\sum\limits_{\alpha}R_{\alpha}^{\text{In}_{li}\text{Se}_{lj}}b_{ljs}^{\dag} a_{lip_{\alpha}}+\sum\limits_{\alpha,\beta}\{[\delta_{\alpha\beta}T_{\pi}^{(\text{In}-\text{Se})_{1}}- \\ (T_{\pi}^{(\text{In}-\text{Se})_{1}}+T_{\sigma}^{(\text{In}-\text{Se})_{1}})R_{\alpha}^{\text{In}_{li}\text{Se}_{lj}} R_{\beta}^{\text{In}_{li}\text{Se}_{lj}}]b_{ljp_{\beta}}^{\dag} a_{lip_{\alpha}}\}\}+\mathrm{H.c.}, \end{aligned}}}  $$

5 $$ { \begin{aligned} H_{ll}^{\text{In}-\text{In}}=\sum\limits_{<\text{In}_{li},\text{In}_{lj}>}\{T_{ss}^{\text{In}-\text{In}}a_{ljs}^{\dag} a_{lis}+T_{sp}^{\text{In}-\text{In}}\sum\limits_{\alpha}R_{\alpha}^{\text{In}_{li}\text{In}_{lj}} a_{ljp_{\alpha}}^{\dag} a_{lis}+ \\ \sum\limits_{\alpha,\beta}\{[\delta_{\alpha\beta}T_{\pi}^{\text{In}-\text{In}}- (T_{\pi}^{\text{In}-\text{In}}+T_{\sigma}^{\text{In}-\text{In}})R_{\alpha}^{\text{In}_{li}\text{In}_{lj}} R_{\beta}^{\text{In}_{li}\text{In}_{lj}}]a_{ljp_{\beta}}^{\dag} a_{lip_{\alpha}}\}\} \\ +\mathrm{H.c.}, \end{aligned}}  $$

6 $$ { \begin{aligned} H_{ll}^{\text{Se}-\text{Se}}=\sum\limits_{<\text{Se}_{li},\text{Se}_{lj}>}\{T_{ss}^{\text{Se}-\text{Se}}b_{ljs}^{\dag} b_{lis}+T_{sp}^{\text{Se}-\text{Se}}\sum\limits_{\alpha}R_{\alpha}^{\text{Se}_{li}\text{Se}_{lj}} b_{ljp_{\alpha}}^{\dag} b_{lis}+ \\ \sum\limits_{\alpha,\beta}\{[\delta_{\alpha\beta}T_{\pi}^{\text{Se}-\text{Se}}- (T_{\pi}^{\text{Se}-\text{Se}}+T_{\sigma}^{\text{Se}-\text{Se}})R_{\alpha}^{\text{Se}_{li}\text{Se}_{lj}} R_{\beta}^{\text{Se}_{li}\text{Se}_{lj}}]b_{ljp_{\beta}}^{\dag} b_{lip_{\alpha}}\}\} \\ +\mathrm{H.c.}, \end{aligned}}  $$

and

7 $$ { \begin{aligned} H_{ll}^{(\text{In}-\text{Se})_{2}}=\sum\limits_{<\text{In}_{li},\text{Se}_{lj'}>}\{T_{ss}^{(\text{In}-\text{Se})_{2}}b_{lj's}^{\dag} a_{lis}+T_{sp}^{(\text{In}-\text{Se})_{2}}\sum\limits_{\alpha}R_{\alpha}^{\text{In}_{li}\text{Se}_{lj'}} \\ b_{lj'p_{\alpha}}^{\dag} a_{lis}+T_{ps}^{(\text{In}-\text{Se})_{2}}\sum\limits_{\alpha}R_{\alpha}^{\text{In}_{li}\text{Se}_{lj'}}b_{lj's}^{\dag} a_{lip_{\alpha}}+\sum\limits_{\alpha,\beta}\{[\delta_{\alpha\beta}T_{\pi}^{(\text{In}-\text{Se})_{2}}- \\ (T_{\pi}^{(\text{In}-\text{Se})_{2}}+T_{\sigma}^{(\text{In}-\text{Se})_{2}})R_{\alpha}^{\text{In}_{li}\text{Se}_{lj'}} R_{\beta}^{\text{In}_{li}\text{Se}_{lj'}}]b_{lj'p_{\beta}}^{\dag} a_{lip_{\alpha}}\}\}+\mathrm{H.c.} \end{aligned}}  $$

include the hopping terms between the nearest-neighbor In-Se, In-In, Se-Se, and next-nearest In-Se pairs within the same sublayer *l*, respectively. $T_{ss/sp/ps}^{\mathrm {X}}$ is the hopping integral for the *ss*/*sp*/*ps* orbitals between the corresponding pair X, while $T_{\pi (\sigma)}^{\mathrm {X}}$ is that for the parallel *p* and *p* orbitals perpendicular to (lying along) the hopping vector $R_{\alpha }^{\mathrm {X}}$ [57]. For example

8 $$\begin{array}{@{}rcl@{}} R_{\alpha}^{(\text{In}-\text{Se})_{1}}=\frac{\mathrm{\mathbf{R}}_{\text{Se}_{lj}}-\mathrm{\mathbf{R}}_{\text{In}_{li}}} {|\mathrm{\mathbf{R}}_{\text{Se}_{lj}}-\mathrm{\mathbf{R}}_{\text{In}_{li}}|}\cdot \hat{\alpha}, \end{array} $$

where $\phantom {\dot {i}\!}\mathrm {\mathbf {R}}_{{\text {In}_{li}}/{\text {Se}_{lj}}}$ is the position vector for In_*li*_/Se_*lj*_, $\hat {\mathbf {\alpha }}$ is a unit vector along *α*.

9 $$\begin{array}{@{}rcl@{}} H_{ll'}=H_{ll'}^{(\text{In}-\text{In})_{1}}+H_{ll'}^{\text{In}-\text{Se}}+H_{ll'}^{(\text{In}-\text{In})_{2}}, \end{array} $$

in which [34]

10 $$ { \begin{aligned} H_{ll'}^{({\text{In}-\text{In}})_{1}}=\sum\limits_{i}\{T_{ss}^{({\text{In}-\text{In}})_{1}}a_{l'is}^{\dag} a_{lis}+T_{sp}^{({\text{In}-\text{In}})_{1}}\sum\limits_{\alpha}R_{\alpha}^{\text{In}_{li}\text{In}_{l'i}} a_{l'ip_{\alpha}}^{\dag} a_{lis}+ \\ \sum\limits_{\alpha,\beta}\{[\delta_{\alpha\beta}T_{\pi}^{({\text{In}-\text{In}})_{1}}- (T_{\pi}^{({\text{In}-\text{In}})_{1}}+T_{\sigma}^{({\text{In}-\text{In}})_{1}})R_{\alpha}^{\text{In}_{li}\text{In}_{l'i}} R_{\beta}^{\text{In}_{li}\text{In}_{l'i}}] \\ a_{l'ip_{\beta}}^{\dag} a_{lip_{\alpha}}\}\}+\mathrm{H.c.}, \end{aligned}}  $$

11 $$ { \begin{aligned} H_{ll'}^{\text{In}-\text{Se}}=\sum\limits_{<\text{In}_{li},\text{Se}_{l'j}>}\{T_{ss}^{\text{In}-\text{Se}}b_{l'js}^{\dag} a_{lis}+T_{sp}^{\text{In}-\text{Se}}\sum\limits_{\alpha}R_{\alpha}^{\text{In}_{li}\text{Se}_{l'j}} \\ b_{l'jp_{\alpha}}^{\dag} a_{lis}+T_{ps}^{\text{In}-\text{Se}}\sum\limits_{\alpha}R_{\alpha}^{\text{In}_{li}\text{Se}_{l'j}}b_{l'js}^{\dag} a_{lip_{\alpha}}+\sum\limits_{\alpha,\beta}\{[\delta_{\alpha\beta}T_{\pi}^{\text{In}-\text{Se}}- \\ (T_{\pi}^{\text{In}-\text{Se}}+T_{\sigma}^{\text{In}-\text{Se}})R_{\alpha}^{\text{In}_{li}\text{Se}_{l'j}} R_{\beta}^{\text{In}_{li}\text{Se}_{l'j}}]b_{l'jp_{\beta}}^{\dag} a_{lip_{\alpha}}\}\}+\mathrm{H.c.}, \end{aligned}}  $$

and

12 $$ { \begin{aligned} H_{ll'}^{({\text{In}-\text{In}})_{2}}=\sum\limits_{i}\{T_{ss}^{({\text{In}-\text{In}})_{2}}a_{l'js}^{\dag} a_{lis}+T_{sp}^{({\text{In}-\text{In}})_{2}}\sum\limits_{\alpha}R_{\alpha}^{\text{In}_{li}\text{In}_{l'j}} a_{l'jp_{\alpha}}^{\dag} a_{lis}+\\ \sum\limits_{\alpha,\beta}\{[\delta_{\alpha\beta}T_{\pi}^{({\text{In}-\text{In}})_{2}}- (T_{\pi}^{({\text{In}-\text{In}})_{2}}+T_{\sigma}^{({\text{In}-\text{In}})_{2}})R_{\alpha}^{\text{In}_{li}\text{In}_{l'j}} R_{\beta}^{\text{In}_{li}\text{In}_{l'j}}] \\ a_{l'jp_{\beta}}^{\dag} a_{lip_{\alpha}}\}\}+\mathrm{H.c.} \end{aligned}}  $$

include the hopping terms between the nearest-neighbor In-In, In-Se, and next-nearest In-In pairs between sublayers *l* and *l*^′^, respectively. If a perpendicular electric field along *z*-axis is applied to the monolayer InSe, its effects can be introduced by a modification of the on-site orbtial energies of In and Se ions, that is,

13 $$\begin{array}{@{}rcl@{}} \varepsilon'=\varepsilon+eE_{z}z, \end{array} $$

where *e* is the electron charge and *E*_*z*_ is the strength of the perpendicular electric field. The perpendicular electric field can be achieved by adding top and bottom gates to the monolayer InSe. Moreover, two insulating layers are inserted between the monolayer InSe and gates to eliminate the electric current along *z*-axis. As a result, the electric field strength can be tuned by varying the gating voltage.

By transforming the tight-binding Hamiltonian in Eq. (1) into the **k** space and then diagonalizing it, energy bands *E*(**k**) of monolayer InSe without or with a perpendicular electric field can be obtained conveniently, where **k** is wave vector. At the same time, the coefficient of eigenvector **C**_*n***k**_(*o*) at band *n*, orbital *o*, and wave vector **k** can also be achieved.

## Numerical Results and Discussions

The lattice parameters of monolayer InSe in Fig. 1a and b are taken as *a*=3.953 Å, *d*=2.741 Å, and *D*=5.298 Å, which are obtained by the local density approximation [30]. The on-site and hopping energies in the tight-binding Hamiltonian Eq. (1) are given in Table 1, which are fitted by the density functional theory data with scissor correction [34]. Although only the numerical results of the monolayer InSe are given here, qualitatively similar results have also been found in the bilayer InSe and the bulk InSe. For conciseness, they are not presented in this letter.

**Table 1:** Parameters (eV) of the tight-binding Hamiltonian in Eq. (1)

$\varepsilon _{\text {In}_{s}}\phantom {\dot {i}\!}$	− 7.174	$\phantom {\dot {i}\!}\varepsilon _{\text {In}_{p_{x}}}=\varepsilon _{\text {In}_{p_{y}}}$	− 2.302
$\phantom {\dot {i}\!}\varepsilon _{\text {In}_{p_{z}}}$	1.248	$\phantom {\dot {i}\!}\varepsilon _{\text {Se}_{s}}$	− 14.935
$\phantom {\dot {i}\!}\varepsilon _{\text {Se}_{p_{x}}}=\varepsilon _{\text {Se}_{p_{y}}}$	− 7.792	$\varepsilon _{\text {Se}_{p_{z}}}\phantom {\dot {i}\!}$	− 7.362
$T_{ss}^{(\text {In}-\text {Se})_{1}}$	0.168	$T_{sp}^{(\text {In}-\text {Se})_{1}}$	2.873
$T_{ps}^{(\text {In}-\text {Se})_{1}}$	− 2.144	$T_{\pi }^{(\text {In}-\text {Se})_{1}}$	1.041
$T_{\sigma }^{(\text {In}-\text {Se})_{1}}$	1.691	$T_{ss}^{\text {In}-\text {In}}$	− 0.200
$T_{sp}^{\text {In}-\text {In}}$	− 0.137	$T_{\pi }^{\text {In}-\text {In}}$	− 0.433
$T_{\sigma }^{\text {In}-\text {In}}$	− 1.034	$T_{ss}^{\text {Se}-\text {Se}}$	− 1.345
$T_{sp}^{\text {Se}-\text {Se}}$	− 0.800	$T_{\pi }^{\text {Se}-\text {Se}}$	− 0.148
$T_{\sigma }^{\text {Se}-\text {Se}}$	− 0.554	$T_{ss}^{(\text {In}-\text {Se})_{2}}$	0.821
$T_{sp}^{(\text {In}-\text {Se})_{2}}$	0.156	$T_{ps}^{(\text {In}-\text {Se})_{2}}$	− 0.294
$T_{\pi }^{(\text {In}-\text {Se})_{2}}$	0.003	$T_{\sigma }^{(\text {In}-\text {Se})_{2}}$	− 0.455
$T_{ss}^{({\text {In}-\text {In}})_{1}}$	− 0.780	$T_{sp}^{({\text {In}-\text {In}})_{1}}$	− 4.964
$T_{\pi }^{({\text {In}-\text {In}})_{1}}$	− 0.681	$T_{\sigma }^{({\text {In}-\text {In}})_{1}}$	− 4.028
$T_{ss}^{\text {In}-\text {Se}}$	0.574	$T_{sp}^{\text {In}-\text {Se}}$	− 0.651
$T_{ps}^{\text {In}-\text {Se}}$	− 0.148	$T_{\pi }^{\text {In}-\text {Se}}$	0.100
$T_{\sigma }^{\text {In}-\text {Se}}$	0.343	$T_{ss}^{({\text {In}-\text {In}})_{2}}$	− 0.238
$T_{sp}^{({\text {In}-\text {In}})_{2}}$	− 0.048	$T_{\pi }^{({\text {In}-\text {In}})_{2}}$	− 0.020
$T_{\sigma }^{({\text {In}-\text {In}})_{2}}$	− 0.151		

Figure 1c shows the energy band of the monolayer InSe. The conduction bands around point Γ display parabola-like energy dispersion, which are similar to that of other normal semiconductors. However, the band structure along Γ−K is slightly asymmetrical with that along Γ−M. And the lowest two conduction bands crossing each other along both these two directions, as indicated by the red cycles. In contrast to the conduction bands, the highest valence band is flat but slightly inverted around point Γ, forming an interesting Mexican hat-like structure. Therefore, monolayer InSe is an indirect band gap semiconductor, which is quite different from that of bulk InSe since it is a direct band gap semiconductor. The energy gap of monolayer InSe can be obtained by $E_{\mathrm {g}}^{\text {id}}=E_{\mathrm {C}}-E_{\mathrm {A}}=2.715$ eV, which is much enlarged by comparing with that of bulk InSe $E_{\mathrm {g}}^{\mathrm {d}}=1.27$ eV [34]. However, the other valence bands show normal parabola-like energy dispersion.

**Fig. 2:**
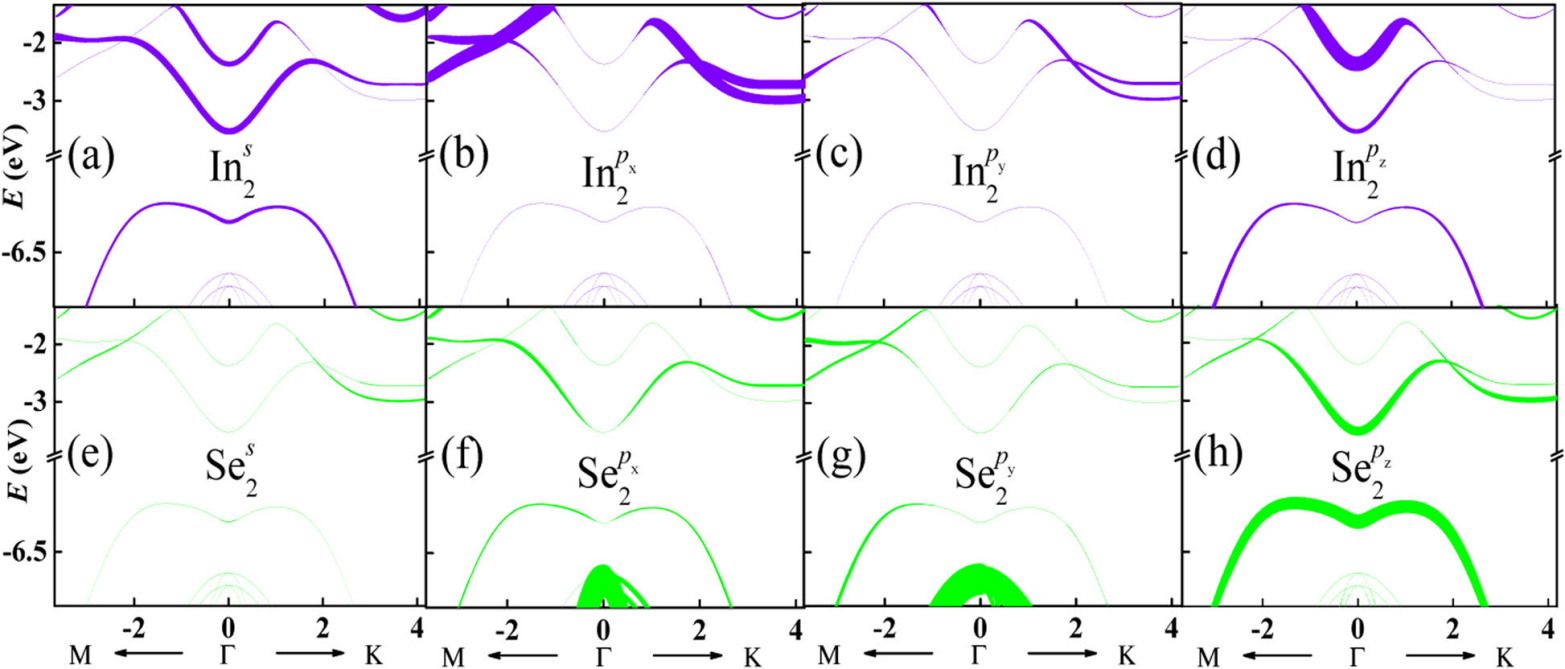
(Color online) Orbital decompositions for the energy band of monolayer InSe. Thicker lines indicate a more dominant contribution. Only In and Se ions in sublayer 2 are selected as examples since the two sublayers of the monolayer InSe with mirror symmetry along *z*-axis (**a**–**h**)

In order to comprehend the energy band of monolayer InSe shown in Fig. 1c, the orbital decomposition |**C**_*n***k**_(*o*)|^2^ for the energy band is given in Fig. 2. As the two sublayers of the monolayer InSe is symmetrical along *z*-axis, the ions in different sublayers have the same orbital contributions to the energy band. Here, In and Se ions in sublayer 2, as shown in Fig. 1b, are taken as examples. The upper panels indicate orbital contributions from In ions while the down panels represent those of Se ions. The thickness of lines is proportional to normalized orbital contribution. It can be seen that the lowest conduction band around point Γ is contributed firstly from *p*_*z*_ orbital of Se ion and then *s* orbital of In ion. The second conduction band around K point dominantly originates from *p*_*x*_ orbital of In ion and then *p*_*z*_ orbital of Se ion. However, the highest valence band is principally contributed from *p*_*z*_ orbital of Se ion. The other valence bands result from both *p*_*x*_ and *p*_*y*_ orbitals of Se ion. These results are consistent with those results obtained by the DFT calculations [34].

**Fig. 3:**
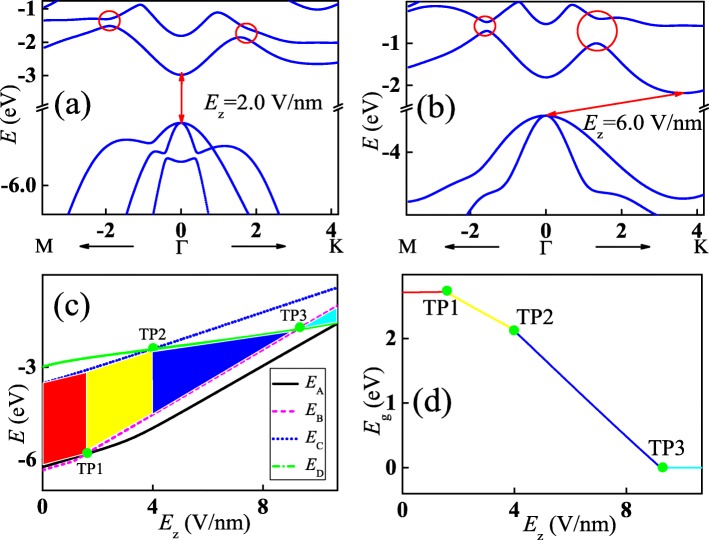
(Color online) Energy bands of the perpendicular electric field-modulated monolayer InSe at different strengths *E*_*z*_=2.0 V/nm(**a**) and 6.0 V/nm (**b**), respectively. Red circles in **a** and **b** mean the opened energy gaps around the crossing points shown in Fig. 1c. **c** Energies at points A (the black solid line), B (the magenta dashed line), C (the blue dotted line), and D (the green dash-dotted line) shown in Fig. 1c as a function of the electric field strength. **d** Global band gap as a function of the strength of the electric field. The yellow line means the direct band gap while the red and blue lines indicate the indirect band gaps

Energy band of the monolayer InSe with a perpendicular electric field along *z*-axis is shown in Fig. 3a. The electric field strength is taken as *E*_*z*_=2.0 V/nm. By comparing with the energy band in Fig. 1c, each conduction and valence band is lifted to the higher energy region as a whole. However, the energy shift of each band is different since its orbital decomposition from the *p*_*z*_ orbital of In and Se ions is different. Position of the maximum value of the highest valence band is changed to point Γ while that of the minimum value of conduction band keeps unchanged. Therefore, the monolayer InSe is transformed into a direct band gap semiconductor. And the energy gap is decreased to $E_{\mathrm {g}}^{\mathrm {d}}=2.61$ eV. Furthermore, the crossings along both Γ−K and Γ−M directions are opened so that energy gaps are generated, as displayed by the red cycles, since the symmetry along *z*-axis is broken by the perpendicular electric field. When the electric field strength is increased to *E*_*z*_=6.0 V/nm, the energy gap at point Γ is decreased but those at the crossings is increased further, as shown in Fig. 3b. Interestingly, position of the minimum value of conduction band is altered from point Γ to that around point K, while that of the maximum value of the highest valence band stay at point Γ. This phenomenon means that the monolayer InSe is transited into indirect band gap semiconductor again and the indirect energy gap of the whole band $E_{\mathrm {g}}^{\text {id}}=1.30$ eV. Similarly, the band gap of monolayer InSe can be controlled by biaxial strain. The band gap ranges from 1.466 to 1.040 eV when the strain is varied from 1 to 4%. In addition, indirect-direct band gap transition is also observed when the monolayer InSe is under uniaxial strain [49]. For the bilayer InSe with a perpendicular electric field, its band gap decreases as the electric field strength increases and it will be closed when the electric field strength is increased to 2.9 V/nm [51].

For the sake of understanding the changing process of electronic structure of monolayer InSe in the presence of a perpendicular electric field more clearly, energies at the wave vectors corresponding to points A, B, C, and D at the band edges shown in Fig. 1c as a function of the strength of electric field are depicted in Fig. 3c. Energies with respect to all these points move upward as the increasing electric field strength, confirming the evolution of the energy bands in Fig. 3a and b. When the electric field strength *E*_*z*_<1.6 V/nm, energy at point A in the valence band is higher than that of point B while the bottom of conduction band locates at point C. Therefore, the electric field-modulated monolayer InSe within this strength range is an indirect band gap semiconductor, as shown by the red area. However, energies with respect to points A and B will cross at TP1, and then energy at point B will be higher than that of point A as the electric field strength is increased further. Simultaneously, the bottom of conduction band keeps unchange until the electric field strength is increased to 4.0 V/nm. As a result, the electric field-modulated monolayer InSe within this strength range is a direct band gap semiconductor, as shown by the yellow area. Similar to the energy crossover between points A and B in the valence band, transit point is also observed in the energies at points C and D in the conduction bands, as indicated by TP2. Energy at point D is lower than that of point C while the top of valence band still stay at point B if only the electric field strength is smaller than 9.23 V/nm. Consequently, the electric field-modulated monolayer InSe is turned into an indirect band gap semiconductor again, as shown by the blue area. Interestingly, energies at point B in the highest valence band and point D in the lowest conduction band will cross at TP3 too, which means that the energy band gap is closed. Moreover, energy at point B will be higher than that of point D when the electric field strength is larger than 9.23 V/nm. Therefore, the lowest conduction band and highest valence band will overlap so that the electric field-modulated monolayer InSe becomes a metal in this case, as shown by the cyan area. The global band gap corresponding to different colored areas in Fig. 3c is plotted in Fig. 3d. The band gap corresponding to the red area is almost independent of the varied electric field strength, as shown by the red line. However, the band gap of the yellow area is decreased linearly with increasing electric field strength. Similar band gap behavior is also found in the blue area but with a larger slope. The band gap is decreased to zero as long as the electric field strength is larger than that at point TP3, as shown by the cyan line. The electric field-modulated band gap behaviors indicate that layered III–VI semiconductors have potential applications in designing novel optical detector and absorbers. Moreover, the spectral response frequency of these devices ranges continuously from the violet light (*ν*≈6.57×10^14^ Hz as *E*_*z*_=1.6 V/nm) to the infrared light (*ν*<3.97×10^14^ Hz as *E*_*z*_>5.18 V/nm).

**Fig. 4:**
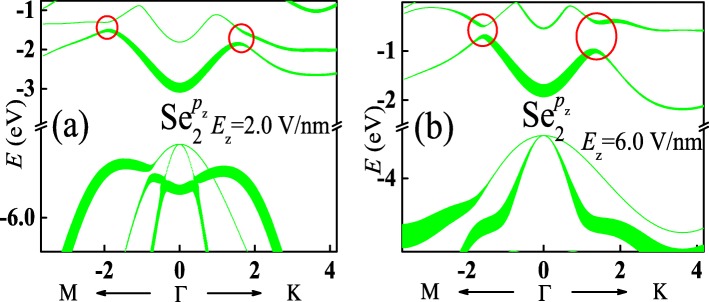
(Color online) **a** and **b** show *p*_*z*_ orbital decomposition of the Se ion in sublayer 2 for the energy bands of the monolayer InSe with a perpendicular electric field shown in Fig. 3a and b, respectively. Thicker lines represent a more important contribution

As well known, electronic characteristics of materials are mainly determined by energy band edges. According to the orbital decomposition for the energy band in Fig. 2, both the conduction and valence band edges of monolayer InSe are dominantly contributed from *p*_*z*_ orbital of Se ion. Therefore, only *p*_*z*_ orbital decompositions of Se ion in sublayer 2 for energy bands shown in Fig. 3a and b are displayed in Fig. 4a and b, respectively. By comparing with Fig. 2h, *p*_*z*_ orbital contribution to the conduction bands is slightly changed. Therefore, the shape of these band structures undergoes little affection. However, the *p*_*z*_ orbital contribution to the valence bands is strongly modified, resulting in the change of shape of these band structures. Moreover, according to the *p*_*z*_ orbital decomposition for the energy band of monolayer InSe with a perpendicular electric field, the relative position of each conduction band keeps unchanged although gaps are opened at band crossings, as indicated by the red cycles. On the contrary, the relative position of each valence band is changed. The energies of the lower valence bands around *Γ* point increase and surpass those of the highest valence band finally, leading to indirect-direct band gap transition.

## Conclusions

Electronic structures of monolayer InSe under the modulation of a perpendicular electric field are investigated. Indirect-direct-indirect band gap transition is found for the monolayer InSe by tuning the electric field strength. Simultaneously, global band gap of this system is decreased monotonously to zero with increasing electric field strength, which means that semiconductor-metal transition is achieved. The evolution of energy band of monolayer InSe in the presence of the perpendicular electric field is clarified by analyzing the energy change of band edge and orbital decomposition for energy band. These results may be helpful in further understanding of the electronic structures of monolayer InSe as well as the designment of monolayer-InSe-based photoelectric devices responding from violet to far-infrared light.

## Data Availability

The datasets supporting the conclusions of this article are included within the article.

## Abbreviations

2D: Two-dimensional
DFT: Density functional theory
TMDs: Transition metal dichalcogenides
